# Supplementary material for: Optical properties of dense lithium in electride phases by first-principles calculations
Source: Sci Rep. 2018 Mar 1;8:3868. doi: 10.1038/s41598-018-22168-1 (PMC5832767; doi:10.1038/s41598-018-22168-1)
Supplement: Supplementary file 1 — Supplementary Material [file 41598_2018_22168_MOESM1_ESM.pdf]

Supplementary Material for "Optical properties of dense lithium in electride phases by first-principles calculations"

Zheng Yu[1], Hua Y. Geng[1], Y. Sun[1], and Y. Chen[2]

[1]*National Key Laboratory of Shock Wave and Detonation Physics, Institute of Fluid Physics, CAEP; P.O.Box 919-102 Mianyang, Sichuan P.R.China, 621900*

[2] *Fracture and Reliability Research Institute, School of Engineering, Tohoku University 6-6-01 Aramakiaoba, Aoba-ku, Sendai 980-8579, Japan*

1. Justification to the computational method

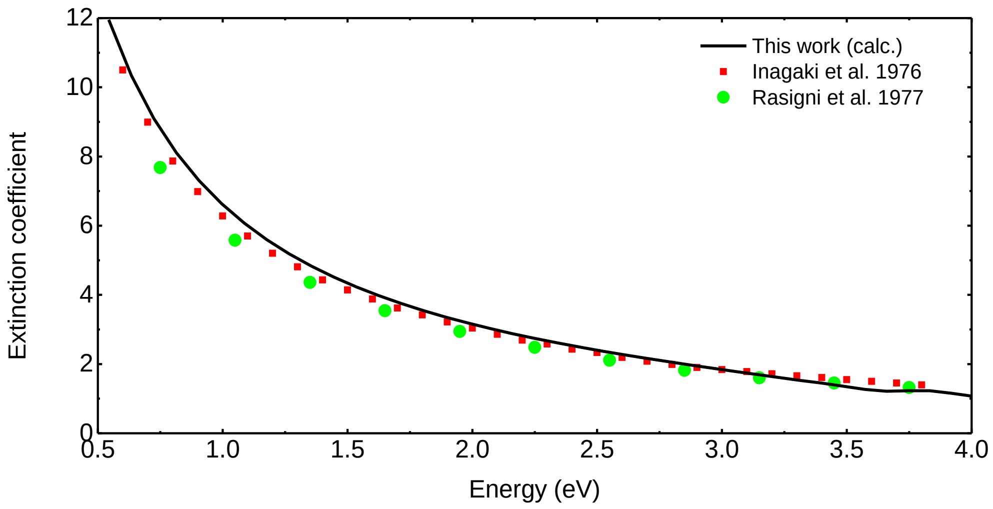

FIG. s1. Comparison of the calculated extinction coefficient of bcc lithium at 0 GPa in this work with the experimental data1,2.

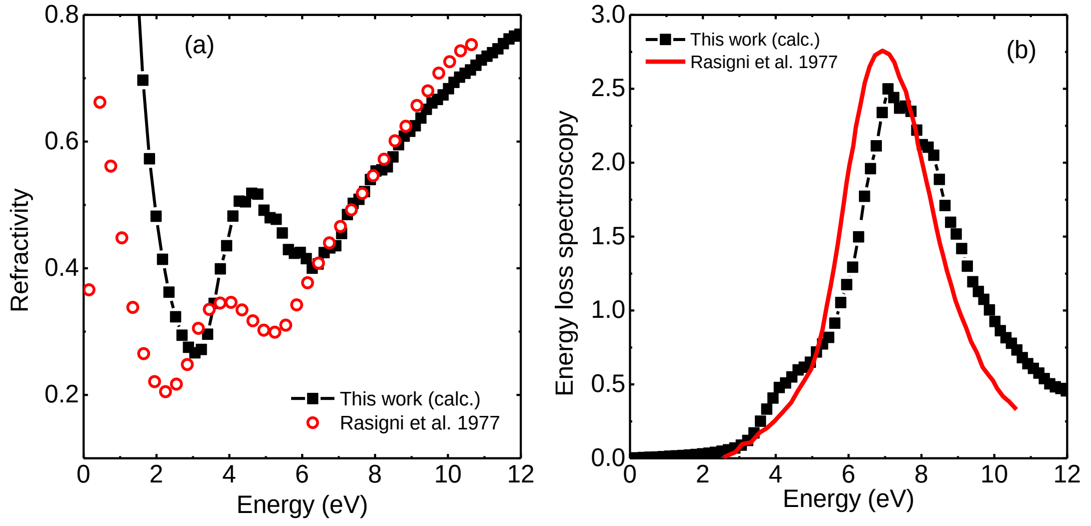

FIG. s2. Comparison of the calculated refractivity and the EELS of bcc lithium at 0 GPa in this work with the experimental results[2].

The validation and accuracy of the method for optical calculations used in this work has been carefully checked with the bcc structure of lithium at ambient conditions. The plasmon frequency of 7.0 eV for bcc lithium by our calculation is in good agreement with the experimental result of 6.73 eV[2]. The calculated energy loss function also matches the experimental spectrum very well, with a main peak of 7.0 eV against the measured 6.9 eV, as shown in Fig. s2(b). The calculated refractive index compares to the experimental data in Fig.2(a). The peaks in our results are slightly shifted to high frequency, but the overall feature agrees with the experimental spectrum. These lend us the confidence that our results are robust and quantitatively accurate.

Still note that the accuracy of optical calculations is in different levels with calculations of total energy. They should be considered as semi-quantitative, especially for the static electric resistivity. The exactly accurate calculation of static optical resistivity needs considerably dense k-points and huge supercells, so that numerous data in extremely low frequency regime could deduce resistivity for $\omega \rightarrow 0$. But this extremely high level of exactness is not required in this work. The data in Figure 11 of the main text is to show the metal-semiconductor-metal transition. We cannot determine whether the metallization of oC24 increases at higher pressure only from the subtle difference of resistivity between 115 GPa and 200 GPa.

In addition, VASP Li pseudopotential needs to be carefully checked. That's why we only consider the pressure range only up to 200 GPa. The latest PAW-PBE VASP pseudopotential of sv_GW with a core radius of 1.500 Bohr was used in this work. We checked its accuracy in a structure of $P4_132$ compared to the results obtained by all-electron full potential augment planewave (FP-LAPW)[3], as Table s1 shows. The pseudopotential of sv_GW shows reliable enough accuracy in calculation of volume of atoms at extreme pressures. The calculation of total energy is certainly affected by the pseudopotential. But the difference is evident only above 200 GPa (see Fig. 1(d) of Reference[3], where "VASP PAW PBE" means the old pseudopotential). The new sv_GW used in this work has better results. The electronic structure was also checked, in agreement with results by FP-LAPW method. In addition, our electronic structure calculation totally coincides with the results by Marques et al.[4]. Therefore, we believe the pseudopotential we used in this work is accurate enough

for our optical research up to 200 GPa.

Table s1: Volumes of atoms calculated by different methods. The sv_GW pseudopotential is the one used in this work. Data of other methods are from Yao[3].

Pressure (GPa)	100	150	200	300	400	500
VASP new sv_GW	0.43%	0.00%	-0.21%	-0.26%	0.06%	0.44%
QE TM PBE	0.00%	0.12%	0.22%	0.37%	0.00%	0.51%
VASP old PAW PBE	1.15%	1.01%	1.03%	2.32%		6.04%
ABINIT GTH PBE	0.20%	0.43%	0.69%	0.61%		1.05%

2. Extra material for cI16

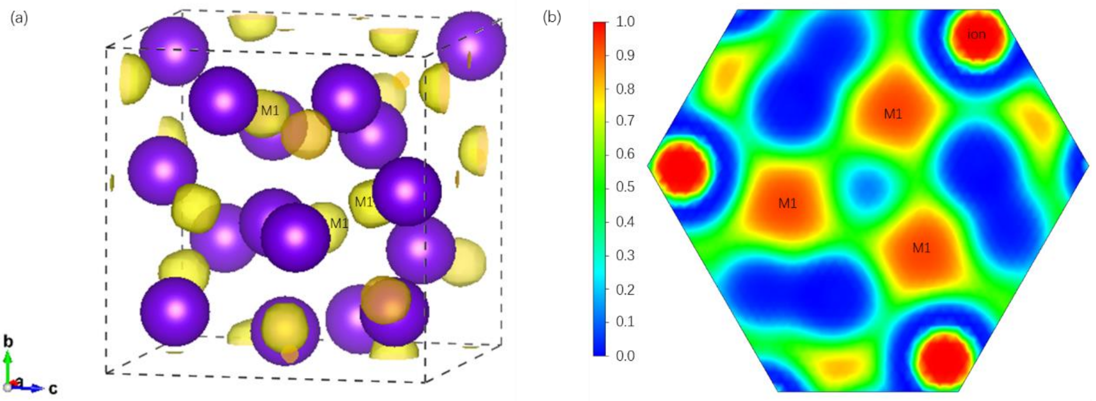

FIG. s3. (a) Structure of cI16 phase with an isosurface of ELF=0.9 at 70 GPa. Lithium ions are colored purple and the ELF isosurface is colored yellow. (b) A 2D display of the plane crossing the three interstitial electrons marked as M1 in (a). cI16 has only one type of ISQ.

The absence of the first peak in Reference[5] (see Fig. 2 of the main text) might be due to the premature cutoff of the extremely low-energy excitations (below 0.5 eV) around the Γ point, whose transition probability, however, is much larger than the high frequency ones. An artificial suppression up to 0.5 eV is evident in Reference[5]. The very close bands near the Fermi surfaces might have been treated as fully degenerate in Alonso's calculations, so their contribution to the interband part below 0.5 eV was ignored.

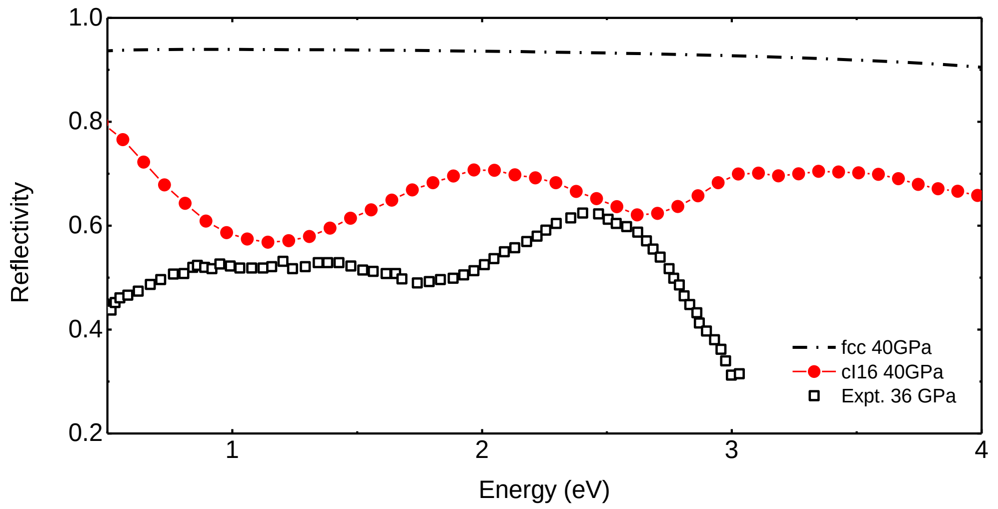

FIG. s4. Comparison of the calculated reflectivity of dense lithium in fcc and cI16 phase at about 40 GPa with experimental data[6]. The agreement between calculations and measurements is qualitative, and the possible reasons for the slight deviation have been discussed in the main text.

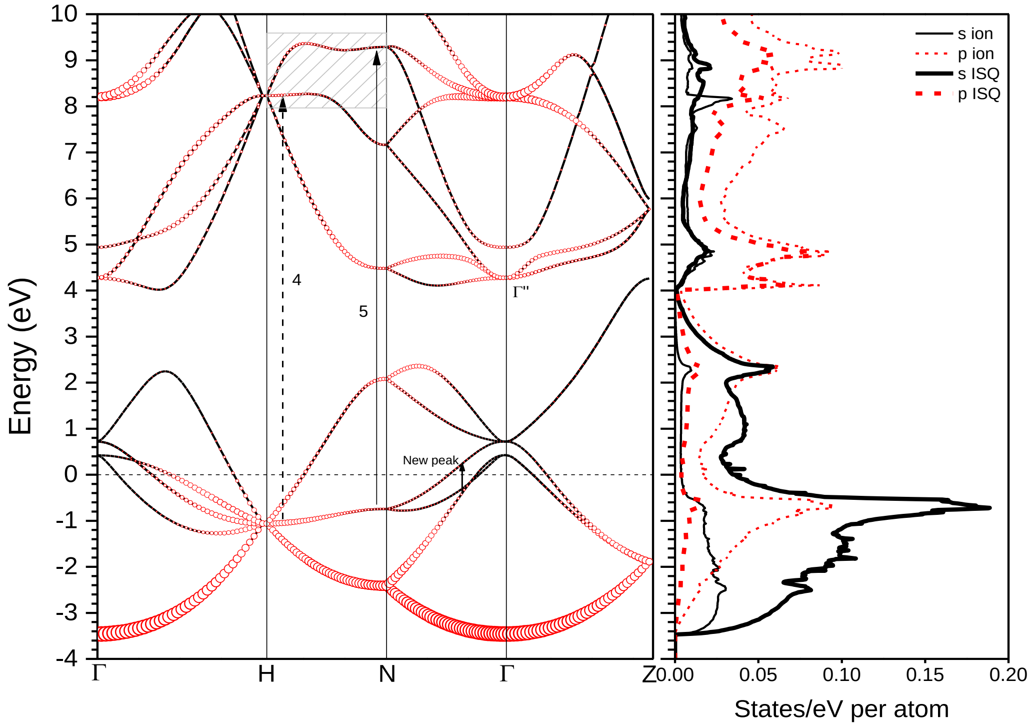

FIG. s5. Calculated electronic band structure and density of states of cI16 at 70 GPa. Other settings are the same as Fig. 1 in the main text. The band marked as Γ′ in Fig. 1 moves up to the position of Γ′′ and forms a triply degenerate state by compression. The shadow rectangle region denotes the parallel bands which enhance the amplitude of the peaks 4 and 5 in the imaginary part of DF. The separating of the bands around Γ point near the Fermi level leads to a new peak at 0.7 eV that is split out from the first main peak as shown in Fig. 2(a) of the main text.

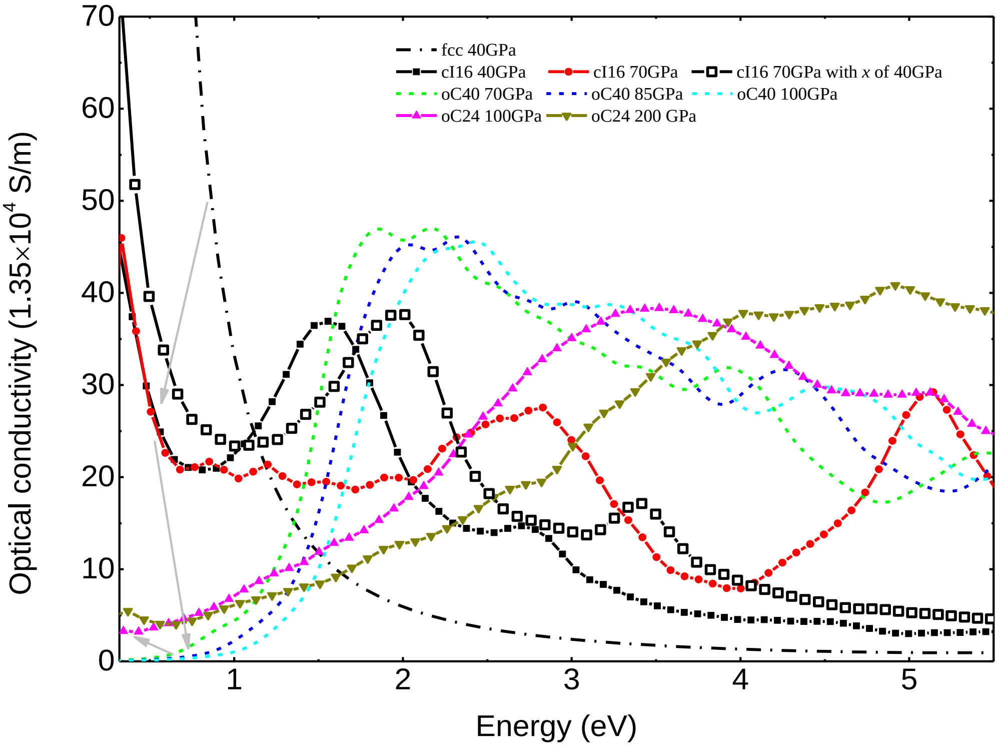

FIG. s6. Variation of the optical conductivity of dense lithium as a function of frequency in three electride phases under increasing pressure. The AC conductivity at low frequency illustrates the optical characteristics of the reentrant transition from metal to semi-metal, to insulator, and then back to a semi-metal phase.

3. Extra material for oC40

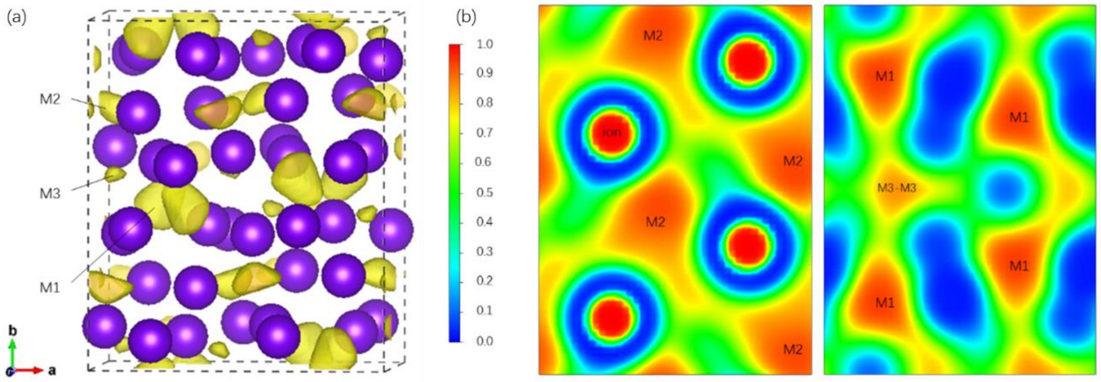

FIG. s7. (a) Structure of oC40 with an isosurface of ELF=0.88 at 70 GPa. Lithium ions are colored purple and the ELF isosurface is colored yellow. (b) 2D displays of the planes perpendicular to (010) crossing the interstitial electrons M1 and M2 marked in (a), respectively. The M3-M3 in the right panel of (b) denotes the chemical bond between adjacent M3s. oC40 has three types of ISQ.

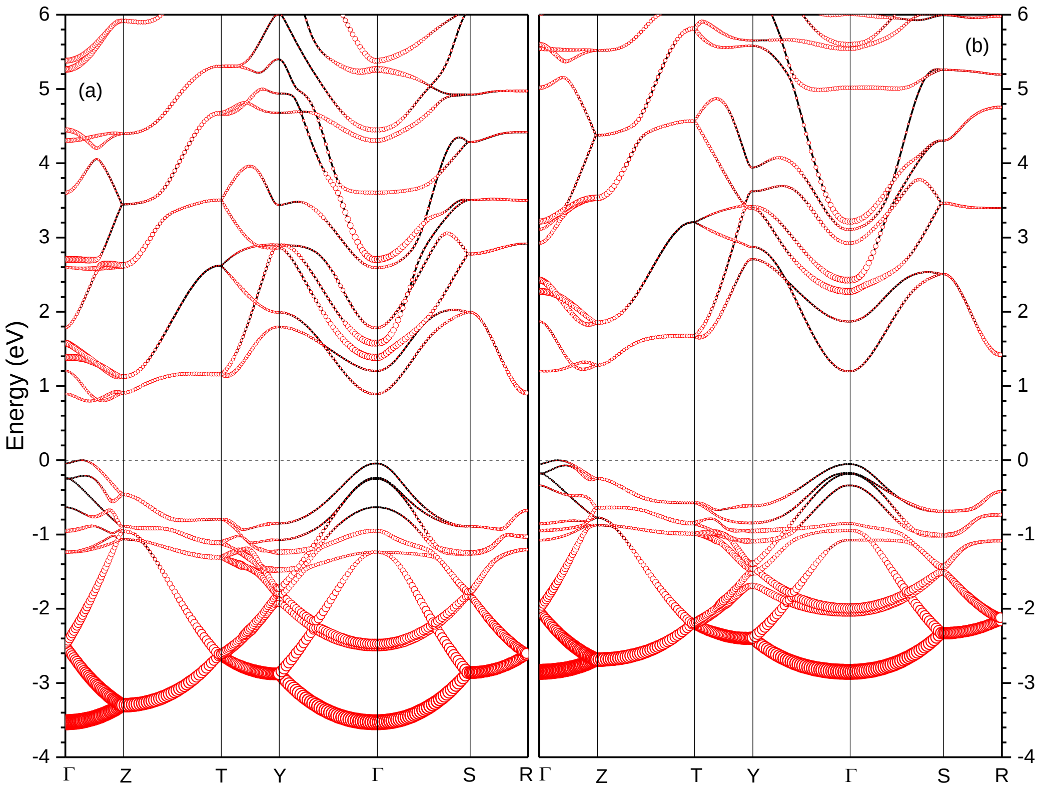

FIG. s8. Comparison of the band structure of oC40 at (a) 70 and (b) 100 GPa. The circle size is proportional to the band *s*-character. The valence bands are counterintuitively narrowed by pressure.

Insulating oC40 and semi-metallic cI16 have totally different symmetries, crystalline and electronic structures, and bonding. It's easy to understand that the electronic bands of oC40 evolve differently with pressure from that of cI16, especially not forming evident degenerate states at Γ point. In addition, as discussions in the manuscript indicate, the cI16 structure is distorted from the bcc structure, where only a few pairs of lithium ions in cI16 are separated by a distance while many others' locations remain unchanged. These unchanged ions also keep relatively unmoved when pressure rises from 40 to 70 GPa. However, the structure of oC40 is optimized more freely and completely at high pressure. All ions will move due to less symmetry constraints when pressure changes, only the distance of very special bonding atoms/quasi-atoms will keep unchanged[7]. Thus, the modification of electronic structure in oC40 is more intuitive, in which the conduction bands not far from the Fermi level are stretched due to different ratios of *2s* contribution. In cI16, due to unique structural distortion and the nesting structure, the electronic structure at Γ point alters abnormally, forming a triply-degenerate state at 70 GPa.

4. Extra material for oC24

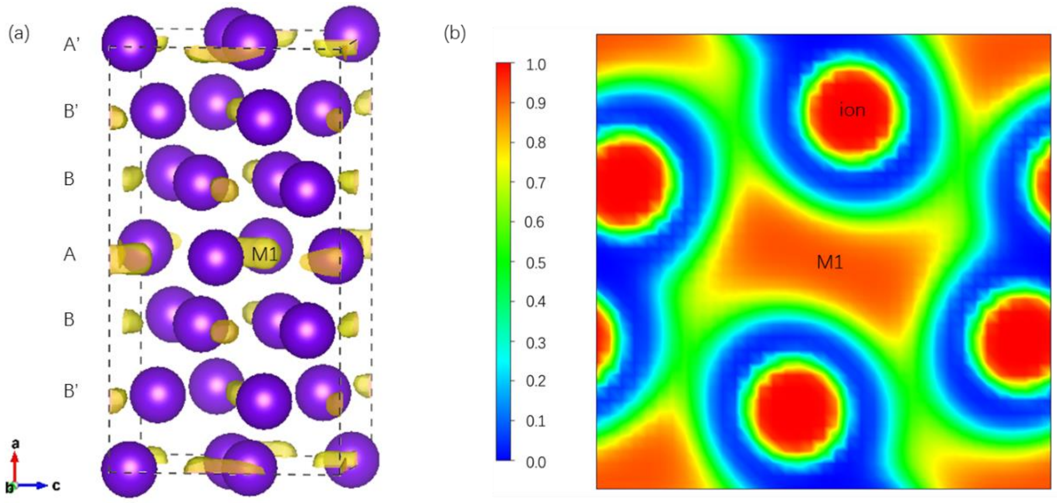

FIG. s9. (a) Structure of oC24 with an isosurface of ELF=0.88 at 100 GPa. Lithium ions are colored purple and the ELF isosurface is colored yellow. (b) A 2D display of the plane perpendicular to (100) crossing the interstitial electrons M1 as marked in (a). oC24 has two types of ISQ.

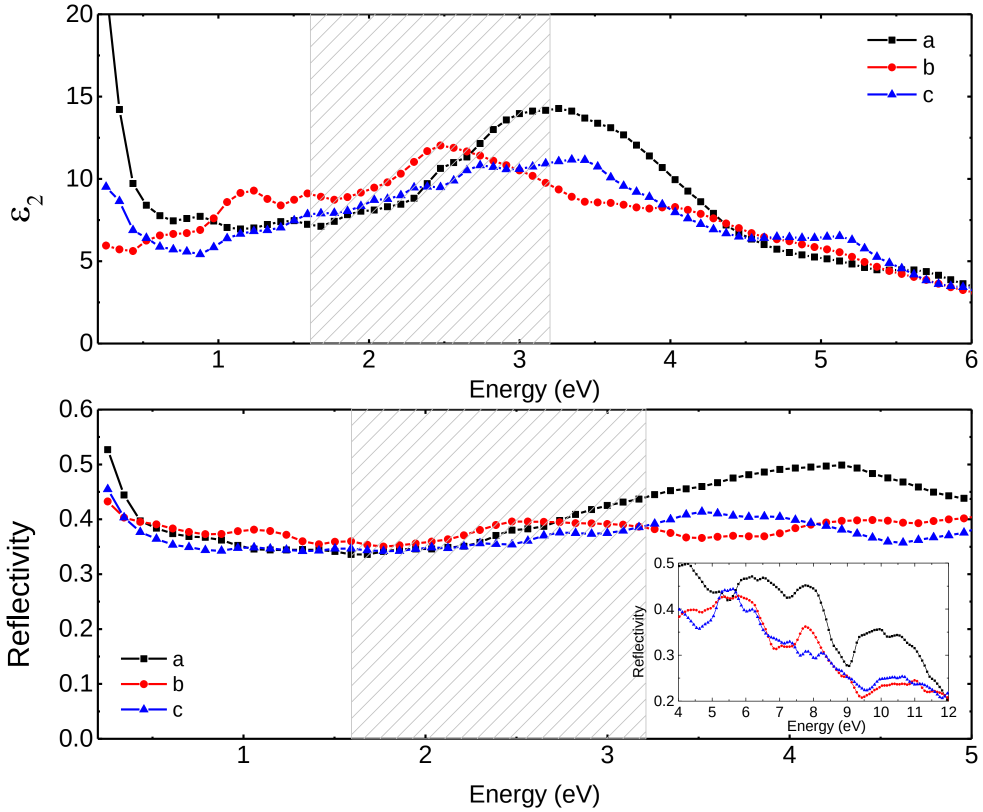

FIG. s10. The calculated anisotropy in the optical properties of oC24 at 100 GPa: (a) the imaginary part of DF; (b) the reflectivity. The anisotropy is weak in the visible light range (1.6-3.2 eV), but becomes noticeable in the ultraviolet regime.

oC24 phase inherits a layered structure from oC40, but has a higher symmetry, with a centrosymmetric distribution of atoms within each layer. Layers A′ and B′ are just another layer

of A and B with relative shifts. oC24 has two non-equivalent interstitial electronic localization positions on layer A and B (ELF = 0.921 and 0.908 at 115 GPa), respectively. This structural feature accounts for the observed optical anisotropy.

The interband contribution to DF from electronic transitions with light polarization along a axis in the low energy region is much larger than along the other two directions, though this anisotropy disappears gradually under further compression. By the analysis in the main text, the first peak in the imaginary DF occurs within the cone around Γ point and this anisotropy represents an uneven feature of the cone structure. The actual momenta of the second peak differ in the three directions. It is at 3.2 eV along a axis, at 2.5 eV along b axis, and with two smaller peaks replacing the main one along c axis at 2.7 and 3.4 eV, respectively. However, the anisotropy in reflectivity is weak in infrared and visible light regime. But it becomes noticeable from 3 to 12 eV, as can be seen in Fig. s10. The reflectivity at 4 eV along a axis is about 0.1 higher than that along b and c axis, which becomes much more disparate at 7 eV and 10 eV.

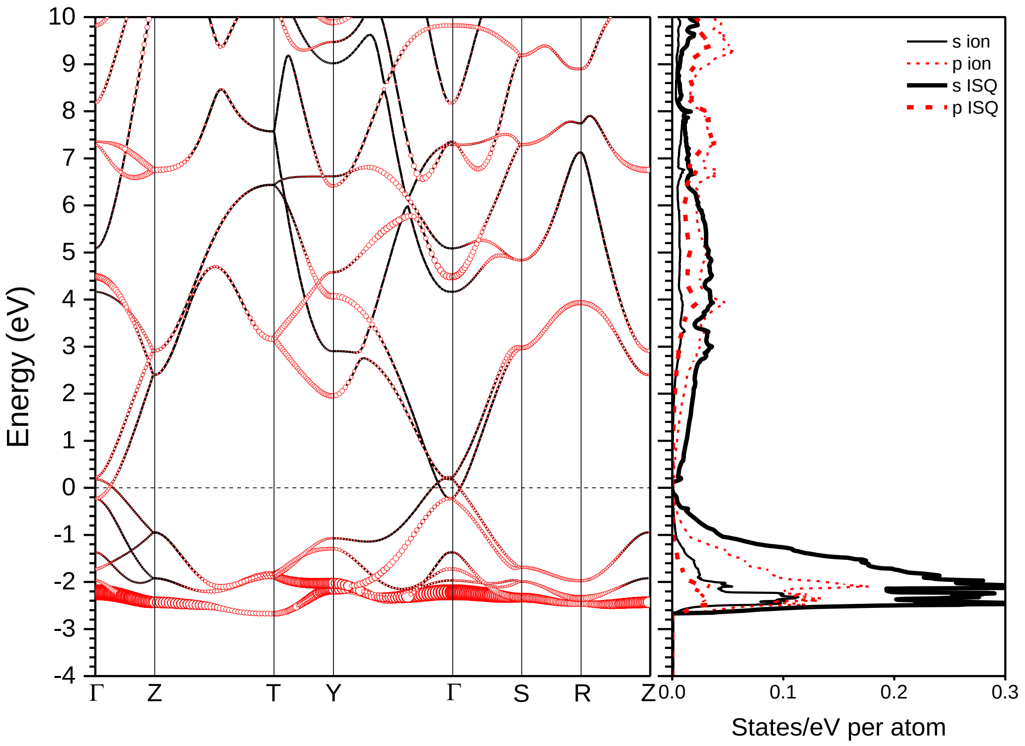

FIG. s11. Calculated electronic band structure and density of states of oC24 at 200 GPa. The main peaks of the *2s* and *2p* component overlap with each other exactly. The valence bands are consequently further narrowed, with striking localization of both *s* and *p* orbitals. But the cone structure is almost the same as oC24 at 100 GPa.

The local details of electronic structure of oC24 near the Fermi surface actually changes subtly with pressure, as shown in FIG 9 and FIG S11. Its cone feature has more area across the Fermi surface and the corresponding energy bands around Γ point also moves relatively. Although the metallization of oC24 could change subtly, it remains weakly metallic up to 200 GPa.

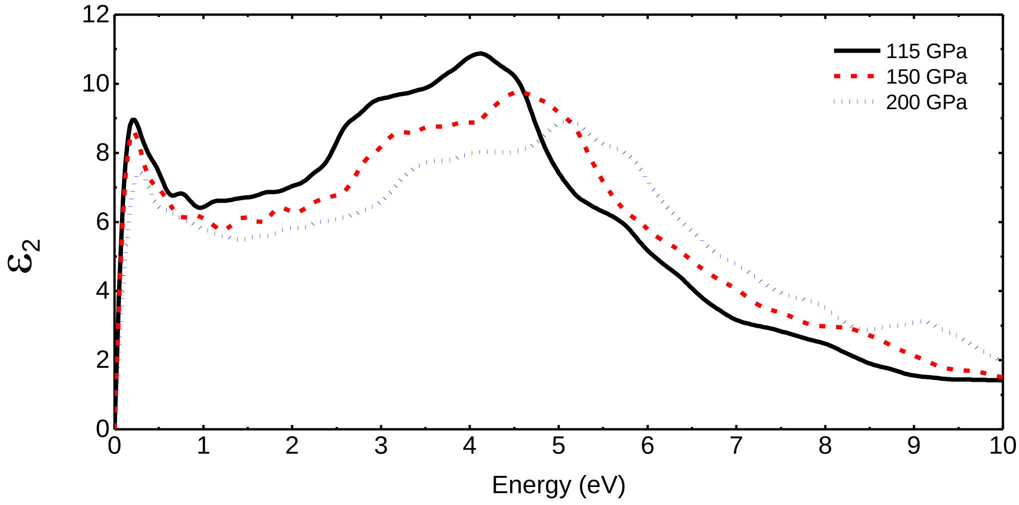

FIG. s12. Variation of the spherically averaged imaginary part of DF in oC24 at 115, 150, and 200 GPa, respectively.

5. Extra data of optical properties

Table s2. The reflectivity, refractive index, and absorption coefficient of high-pressure electride phases of lithium at pressures from 40 to 200 GPa at a frequency of 2, 3 and 4 eV, respectively. Note that oC24 is the most transparent one in the visible light range, even though it is metallic.

2 eV	pressure (GPa)	Reflectivity	Refractive index	Absorption coefficient
fcc	40	0.94	0.34	8.8
cI16	40	0.71	1.5	7.6
	70	0.45	2.2	4.8
	70 (x=0.048)	0.60	2.6	7.2
oC40	70	0.53	3.9	6.1
	85 (along *a* axis)	0.56	4.2	6.6
	85 (along *b* axis)	0.42	3.8	3.6
	85 (along *c* axis)	0.53	4.6	5.7
	100	0.49	4.4	4.4
oC24	100	0.35	3.4	2.6
	200	0.31	3.1	2.0

3 eV	pressure (GPa)	Reflectivity	Refractive index	Absorption coefficient
fcc	40	0.92	0.15	7.9
cI16	40	0.70	0.70	7.6
	70	0.54	1.6	8.0
	70 (x=0.048)	0.56	1.0	6.9
oC40	70	0.50	2.2	8.1
	85 (along *a* axis)	0.51	2.4	8.5
	85 (along *b* axis)	0.39	2.6	6.1
	85 (along *c* axis)	0.53	2.4	8.9
	100	0.46	2.6	7.5
oC24	100	0.40	2.9	6.0
	200	0.32	3.1	3.8

4 eV	pressure (GPa)	Reflectivity	Refractive index	Absorption coefficient
fcc	40	0.90	0.10	6.8
cI16	40	0.65	0.38	6.3
	70	0.30	0.85	4.8
	70 (x=0.048)	0.61	0.59	7.4
oC40	70	0.49	1.6	9.6
	85 (along *a* axis)	0.52	1.3	9.2
	85 (along *b* axis)	0.38	2.2	7.9
	85 (along *c* axis)	0.42	1.7	8.5
	100	0.44	1.7	8.8
oC24	100	0.43	2.0	8.9
	200	0.36	2.6	7.2

6. ELF information on the three electride phases

Table s3: Bader charge and ELF analysis of dense lithium.

Structure	fcc	cI16		oC40		oC24	
Pressure (GPa)	40	40	70	70	100	100	200
Charge of each ion	2.28	2.29	2.32	2.32	2.34	2.35	2.39
ISQ charge occupation (from Bader analysis)	23.8%	23.6%	22.8%	22.7%	22.0%	21.8%	20.4%
Maximum of ELF (interstitial)	0.861	0.914	0.94	0.961	0.959	0.921	0.918
Interstitial space occupation (ELF>0.9)	0.00%	0.49%	2.53%	2.46%	2.90%	0.65%	0.47%
Interstitial space occupation (ELF>0.8)	2.39%	7.26%	8.94%	9.26%	9.37%	6.58%	4.88%

Table s3 is to aid us understand the electron distribution in the electride phases of lithium. The occupation of interstitial space with high ELF is calculated to represent the degree of the electron localization. The localization is greatly enhanced for cI16 from 40 to 70 GPa, as the occupation volume with ELF greater than 0.9 increases 5 times compared to other two phases. This striking change in the electron distribution results in a structural distortion of cI16, as discussed in the main text. The localization of oC24 is weakened at high pressure, which shows a weak metallic behavior.

7. Extra discussion

The *2p* contribution to the orbitals around ions certainly increases with pressure from 40 to 200 GPa and it plays a significant role in dense lithium. With the increase of *2p* contribution, the $p\pi$ bonding in cI16 becomes more saturated, allowing more valence electrons to localize at interstitial space. Thus, the structure of cI16 is gradually distorted with the increment of the internal coordinate *x*, creating more room in the interstitial region. Due to the nesting structure in cI16, there exists an energy gap between the highest not fully occupied valence band and the lowest conduction band. The gap becomes bigger with pressure and finally the structure stabilized by the $p\pi$ bonding is not stable anymore. The low-symmetric insulating oC40 has a lower enthalpy above 66 GPa due to the saturated multi-centered chemical bonding and the bonding between interstitial electrons of E1[7-9]. Under further compression the structure alters subtly, and the energy gap becomes larger, because the *2s* contribution at Γ-Z, Z-T, and T-Y makes the lowest conduction bands rises faster than the highest valence bands with the 2p contribution. As atomic structure becomes harder, the increasing of *2p* contribution becomes slower and it is not the main causes of these structural changes. The repulsion of interstitial electrons is more evident, making the energy reach the value for bond cleavage. Some interstitial electrons tend to be free. As a result, dense lithium transits into the reentrant weak metallic phase oC24. It can be confirmed by that the maximum of ELF in oC40 and oC24 decreases continuously with pressure.

As Figures 1, 6, and 9 in the main text show, the major peaks in the excitation spectra are related to those excitations from the highly occupied *2p* states not far below the Fermi surface to the corresponding *2s* states in conduction bands. As external pressure rises, though the *2p* contribution in valence bands increases, the *2s* states contributes more to the changes of excitation

spectra, because they rise far faster than the *2p* states in band structure, resulting in the movement of the excitation spectra to higher frequencies. Except this, the changes of excitation spectra in cI16 is unique compared to oC40 and oC24, due to its structural distortion as we discussed in the manuscript, which is highly related to the *2p* contribution.

Reference

1 Inagaki, T., Emerson, L. C., Arakawa, E. T. & Williams, M. W. Optical properties of solid Na and Li between 0.6 and 3.8 eV. *Phys. Rev. B* **13**, 2305-2313 (1976).

2 Rasigni, G. & Rasigni, M. Optical constants of lithium deposits as determined from the Kramers-Kronig analysis. *J. Opt. Soc. Am.* **67**, 54-59 (1977).

3 Yao, Y., Tse, J. S., Song, Z. & Klug, D. D. Core effects on the energetics of solid Li at high pressure. *Phys. Rev. B* **79**, 092103, doi:10.1103/PhysRevB.79.092103 (2009).

4 Marques, M. *et al.* Crystal structures of dense lithium: a metal-semiconductor-metal transition. *Phys. Rev. Lett.* **106**, 095502, doi:10.1103/PhysRevLett.106.095502 (2011).

5 Alonso, R., Sharma, S., Ambrosch-Draxl, C., Rodriguez, C. & Christensen, N. E. Linear and nonlinear optical properties of Li under pressure. *Phys. Rev. B* **73**, 064101 (2006).

6 Goncharov, A. F., Struzhkin, V. V., Mao, H.-k. & Hemley, R. J. Spectroscopic evidence for broken-symmetry transitions in dense lithium up to megabar pressures. *Phys. Rev. B* **71**, 184114, doi:10.1103/PhysRevB.71.184114 (2005).

7 Miao, M. s., Hoffmann, R., Botana, J., Naumov, I. I. & Hemley, R. J. Quasimolecules in Compressed Lithium. *Angew. Chem.* **129**, 992-995 (2017).

8 Naumov, I. I. & Hemley, R. J. Origin of transitions between metallic and insulating states in simple metals. *Phys. Rev. Lett.* **114**, 156403, doi:10.1103/PhysRevLett.114.156403 (2015).

9 Naumov, I. I., Hemley, R. J., Hoffmann, R. & Ashcroft, N. W. Chemical bonding in hydrogen and lithium under pressure. *J. Chem. Phys.* **143**, 064702, doi:10.1063/1.4928076 (2015).
